# Draft Genome Sequence of *Granulicatella* sp. Strain S8, Isolated from a Marine Fish, Seriola quinqueradiata

**DOI:** 10.1128/mra.01352-22

**Published:** 2023-03-13

**Authors:** Myunglip Lee, Adeel Farooq, Joon Bum Jeong, Man-Young Jung

**Affiliations:** a Department of Marine Life Science, Jeju National University, Jeju, Republic of Korea; b Research Institute for Basic Sciences (RIBS), Jeju National University, Jeju, Republic of Korea; c Department of Science Education, Jeju National University, Jeju, Republic of Korea; d Interdisciplinary Graduate Programme in Advance Convergence Technology and Science, Jeju National University, Jeju, Republic of Korea; Montana State University

## Abstract

In this study, *Granulicatella* sp. strain S8 was isolated from the gut of a marine fish, Seriola quinqueradiata, and the draft genome was sequenced. Various genes responsible for pathogenesis, metabolite biosynthesis, defense, and lactic acid production were predicted. The genome sequence of this facultative anaerobe provides insights into its distinctive features.

## ANNOUNCEMENT

The genus *Granulicatella* belongs to the family *Carnobacteriaceae* and consists of three species (Granulicatella adiacens, Granulicatella elegans, and Granulicatella balaenopterae). It was reclassified from the genus *Abiotrophia* (1). The genus *Granulicatella* comprises Gram-positive bacteria isolated from various sources, including the internal organs of a minke whale (2) and patients with acute endocarditis (3, 4). It has been reported that nutritionally variant streptococci (NVS), including G. adiacens and G. elegans, are normal constituents of the human oropharyngeal, gastrointestinal, and urogenital flora (5, 6). This study analyzed the draft genome of *Granulicatella* sp. strain S8, isolated from the gut of a fish, Seriola quinqueradiata.

A marine fish, S. quinqueradiata, was sampled from the sea of Jeju Island in South Korea, and gut content samples were serially diluted to 10^−4^ with phosphate-buffered saline (PBS), and 200 μL of each of the dilutions was spread on lactobacilli de Man, Rogosa, and Sharpe (MRS) agar (MRS; Difco). Afterward, the agar plates were incubated at 30°C for 72 h. Several single colonies were randomly selected to inoculate on the new MRS agar. Finally, strain S8 was isolated on brain heart infusion (BHI) agar at 30°C.

DNA of strain S8 was extracted using the QIAamp DNA minikit (Qiagen). The TruSeq Nano DNA library preparation kit was used to prepare a paired-end DNA library, and genome sequencing was performed using Illumina HiSeqX at Macrogen (Korea). The resulting paired-end reads were 150 × 2 bp in length. Trimmomatic v.0.39 was used to remove adapters and low-quality reads (7), whereas FastQC (http://www.bioinformatics.babraham.ac.uk/projects/fastqc) was used to check the overall quality of the filtered data. Consequent high-quality reads were assembled by SPAdes (v.3.13.0) (8). Genome size was estimated through Jellyfish (https://github.com/gmarcais/Jellyfish), which works by counting k-mer. Genome completion was determined through BUSCO v.5.4.4 analysis (9). Functional annotation of the genome was performed using the NCBI prokaryotic genome annotation pipeline (10).

In total, 14,700,428 reads were generated and subsequently filtered for quality to yield 13,069,158 reads. The genome of the strain S8 is 2,224,566 bp in size with 38.28% GC content, 257,477-bp *N*_50_ value, and average coverage of 150×, while it is comprised of 1,959 protein-coding genes; 9 rRNAs consist of 5S (2 complete, 2 partial), 16S (1 complete, 1 partial), and 23S (3 partial); and 47 tRNA genes (Table 1). This strain was identified based on its 16S rRNA gene phylogeny, which showed the greatest similarity (96.2%) to Granulicatella adiacens (ATCC 49175). Further, a digital DNA-DNA hybridization (dDDH) and average nucleotide identity (ANI) metric comparison of the S8 genome with three publicly available *Granulicatella* genomes was used to identify the distinctiveness of the species. In both cases, the dDDH (25%) and ANI (85.51%) values fell below the thresholds for species boundaries of 70% and 95 to 96%, respectively (11–14). Thus, genome-based metrics and phylogenomic analysis revealed that it is a distinctive species belonging to the genus *Granulicatella*.

**TABLE 1:** Genomic features of *Granulicatella* sp. strain S8

Feature	Value
Genome size (bp)	2,224,566
No. of contigs	26
GC content (%)	38.28
*N*_50_ (bp)	257,477
Total no. of genes	2,045
Total no. of protein-coding sequences	1,959
No. of tRNA	47
No. of rRNAs (5S, 16S, 23S)^*a*^	4, 2, 3 (2, 1, 3)^*a*^
No. of CRISPR/Cas elements	2
Human-pathogenic protein (GenPept accession no.)	50S ribosomal protein L16 (AAV42191)
	30S ribosomal protein S19 (ADB66989)
	50S ribosomal protein S21 (ABP90399)

a Number of partial genes.

Several putative genes for carbohydrate degradation, lactic acid production, and terpenoid backbone biosynthesis were predicted in the S8 genome. In addition, the genome also encodes defense- and stress-related attributes, along with the adaptive immunity comprising of type II and type V CRISPR/Cas systems. Moreover, the S8 genome contains proteins that support the scavenging of reactive oxygen species (ROS), including superoxide dismutase, glutathione peroxidase, and thiol peroxidase, although it lacks catalase.

During facultative anaerobic respiration, the strain is predicted to depend on the activity of flavin-dependent oxidoreductase of the LLM class and anaerobic ribonucleoside-triphosphate reductase. Human-pathogenic protein families were also identified in the S8 genome by PathogenFinder (15).

### Data availability.

The draft genome sequencing data of strain S8 (Taxonomy ID 2967226) is available under NCBI BioProject accession number PRJNA861907. The BioSample and GenBank accession numbers are SAMN29932823 and JANHNZ000000000, respectively. The raw sequencing reads were deposited in the Sequencing Read Archive (SRA) database under accession number SRR20853192. The isolated strain S8 has also been deposited at the Korean Collection for Type Cultures (KCTC) as KCTC 43438 and the Japan Collection of Microorganisms (JCM) as JCM 35064.
